# PI3K induces B-cell development and regulates B cell identity

**DOI:** 10.1038/s41598-018-19460-5

**Published:** 2018-01-22

**Authors:** Hend Abdelrasoul, Markus Werner, Corinna S. Setz, Klaus Okkenhaug, Hassan Jumaa

**Affiliations:** 1 Institute of Immunology, University Medical Center Ulm, 89081 Ulm, Germany; 2 Molecular Biology department, Genetic Engineering and Biotechnology Division, National Research Centre (NRC), 12622 Giza, Egypt; 3 Laboratory of Lymphocyte Signalling and Development, The Babraham Institute, Cambridge, CB22 3AT UK

## Abstract

Phosphoinositide-3 kinase (PI3K) signaling is important for the survival of numerous cell types and class IA of PI3K is specifically required for the development of B cells but not for T cell development. Here, we show that class IA PI3K-mediated signals induce the expression of the transcription factor Pax5, which plays a central role in B cell commitment and differentiation by activating the expression of central B cell-specific signaling proteins such as SLP-65 and CD19. Defective class IA PI3K function leads to reduction in Pax5 expression and prevents B cell development beyond the stage expressing the precursor B cell receptor (pre-BCR). Investigating the mechanism of PI3K-induced Pax5 expression revealed that it involves a network of transcription factors including FoxO1 and Irf4 that directly binds to the Pax5 gene. Together, our results suggest that PI3K signaling links survival and differentiation of developing B cells with B cell identity and that decreased PI3K activity in pre-B cells results in reduced Pax5 expression and lineage plasticity.

## Introduction

The development of B cells is a highly-regulated process with defined stages characterized by cell-surface markers and the recombination status of the *immunoglobulin* (*Ig*) genes^1^. The development along the B-cell lineage requires the coordinated action of a variety of transcription factors, of which Pax5 has emerged to be essential for lineage commitment^2^. Besides inducing transcription of important genes required for B-cell development such as *Cd19*^3^ and *Blnk* (the gene encoding SLP-65; SH2-domain containing protein of 65 kDA, also known as BLNK or Bash)^4^, Pax5 represses transcription of B-lineage inappropriate genes, thereby enforcing B cell character^2^. Accordingly, Pax5-deficient mice have a severe B cell developmental block at the pro-B cell stage with progenitor B cells lacking the ability to proceed with B cell development. Furthermore, inactivation of Pax5 in mature B cells induces their de-differentiation and the ability to enter other hematopoietic lineages^2^.

During B-cell development, successful VDJ recombination of the Ig heavy chain genes *(IgH)* leads to generation of a μ heavy chain (μHC), which is crucial for expression of a precursor B cell receptor (pre-BCR)^5,6^. The pre-BCR complex comprises two μHCs associated with the surrogate light chain (SLC) components VpreB and λ5 and the signal transduction subunits Ig-α and Ig-β^7^. There is large body of evidence that autonomously induced pre-BCR signals are required for cell cycle progression and proliferation of developing B cells^8^. On the other hand, pre-BCR signals are equally required for subsequent pre-B cell differentiation to early immature B cells^9^. Hence, pre-BCR signaling activates two fundamentally different cellular processes, namely proliferation and differentiation.

The differentiation of pre-B cells and the initiation of Ig light chain gene (*IgL*) recombination are induced by pre-BCR dependent activation of SLP-65^9,10^. Expression of SLP-65 in B cells depends entirely on Pax5^4^ and inactivation of the gene encoding SLP-65 blocks B cell development at the pre-B cell stage^11–13^. SLP-65-deficient B cells express increased amounts of pre-BCR on the surface and show enhanced proliferation, suggesting that SLP-65 acts as tumor suppressor^10,11^. Indeed, activation of SLP-65 promotes exit from the cell cycle, down-regulation of pre-BCR expression and the initiation of *IgL* gene recombination by diminishing phosphoinositide-3 kinase (PI3K) activity^9,10^.

It has been shown that signals for B cell survival are mediated by class IA of PI3Ks, which are heterodimers consisting of a catalytic subunit (p110α, p110β or p110δ) that is coupled to one of five regulatory subunits (p85α, p85β, p55α, p55γ, p50α)^14,15^. Class IA PI3Ks become activated upon recruitment to the plasma-membrane by binding to adaptor proteins such as CD19 or B cell adaptor protein (BCAP)^16^. PI3K activity leads to the production of phosphatidylinositol-(3,4,5)-trisphosphate (PtdInsP_3_), which is required for membrane recruitment and subsequent activation of important signaling proteins including AKT (also known as protein kinase B or PKB)^17,18^. The role of class IA PI3K in B cell development was first shown in mice deficient for the regulatory subunit p85α or for the catalytic subunit p110δ^19–23^. However, in these mice B cell development was only slightly blocked at the pre-B cell stage, indicating that B cell development is regulated by the redundant function of several PI3K subunits. In fact, combined inactivation of both p110α and p110δ catalytic subunits (*p110*α^fl/fl^/*δ*^D910A^ x CD2-Cre) results in a severe block at the pre-B cell stage while the pro-B cell numbers are significantly elevated in these mice^24^. Similarly, combined absence of AKT1 and AKT2 during B cell development leads to accumulation of pro-B and pre-B cells, whereas numbers of immature and mature B cells are decreased^25^. Together, these studies indicate that pre-B cells defective for class IA PI3K/AKT signaling are unable to proceed in differentiation^25^ suggesting that PI3K acts downstream of the pre-BCR and that expansion and survival of pro-B cells is independent of PI3K. Interestingly, only B cell development is blocked in the p110α/p110δ-double deficient mice, although both catalytic subunits were defective in early progenitors of both, B and T lymphocytes^24^. The finding that T-cell development was not affected by inactivation of p110α and p110δ suggests that class IA PI3K-dependent-signaling is crucial for activation of B cell-specific signaling cascades that particularly promote B cell development. However, it has recently been suggested that PI3K is activated only by the interleukin-7 receptor (IL-7R) and not by the pre-BCR in pre-B cells^26,27^. This appears to be in sharp contrast to the inevitable role of IL-7R in murine B and T cell development as compared to the dispensable role of PI3K specifically in T cell development.

Here, we explored the activation and function of class IA PI3K during early B cell development and found that it is activated by the pre-BCR and not by IL-7R. Moreover, we show that class IA of PI3K is required for B cell commitment and differentiation as it activates the expression of the B cell transcription factor Pax5.

## Results

### PI3K regulates Pax5 expression

**Figure 1:**
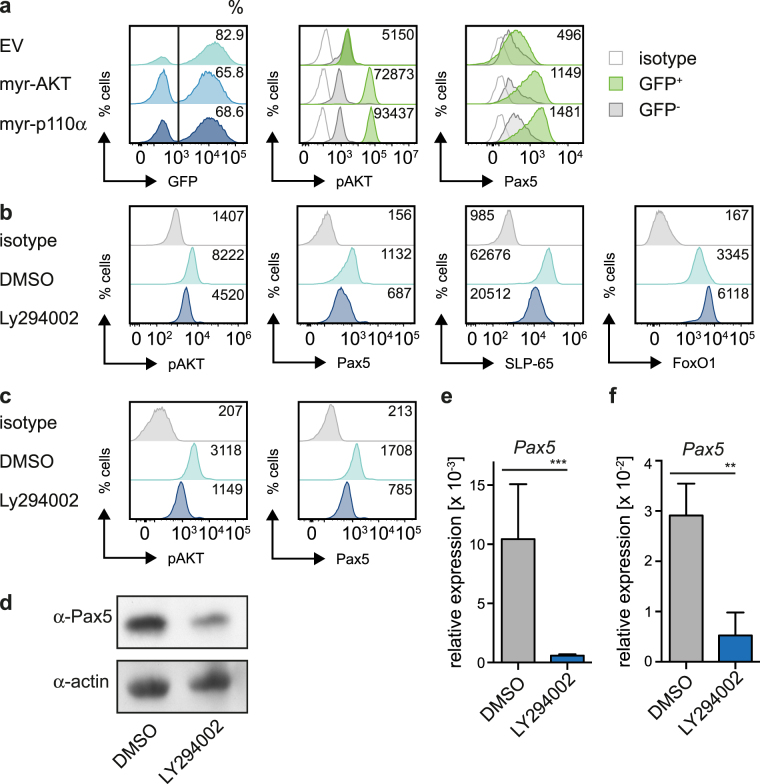
PI3K regulates Pax5 expression. (**a**) Cells from a bone marrow (bm)-derived wildtype (wt) pre-B cell culture were transduced with constitutively active forms of AKT (myr-AKT), p110α (myr-p110α), or as control with empty vector (EV) and analyzed for pAKT and Pax5 expression by intracellular FACS. If not indicated otherwise, numbers in the histograms state the mean fluorescence intensity (MFI) of the respective GFP^+^ populations. (**b**) Cells from a bm-derived wt pre-B cell culture were treated with LY294002 or DMSO for 16 h and analyzed for pAKT, Pax5, SLP-65 and FoxO1 expression by intracellular FACS. (**c**) Cells from a SLP-65-deficient pre-B cell line were treated with LY294002 or DMSO for 12 h and pAKT and Pax5 expression was analyzed by intracellular FACS. (**d**) Murine mature B cells (CD43^−^) were isolated and treated with LY294002 or DMSO for 12 h, lysed and subjected to immunoblot for analysis of Pax5 expression. Actin served as a loading control. For original full-length blots see Fig. S1e. (**e**) Total RNA of SLP-65-deficient cells treated for 12 h with LY294002 or DMSO was isolated. *Gapdh* and *Pax5* mRNA-levels were detected with specific primers by qRT-PCR using the SYBR-Green detection method. Results are shown as mean ± SD of 2 independent experiments, run as duplicates. Statistical significance was calculated using the t-Test. (**f**) Total RNA of murine mature B cells treated with LY294002 or DMSO for 12 h was isolated. *Gapdh* and *Pax5* mRNA-levels were detected with specific primers by qRT-PCR using the SYBR-Green detection method. Results are shown as mean ± SD of 2 independent experiments, run as duplicates. Statistical significance was calculated using the Mann-Whitney Test. Data shown in Fig. 1a–d are representative of at least 3 independent experiments.

Analysis of mice with impaired class IA PI3K in B/T lymphocyte progenitors revealed that B cell development is selectively blocked at the pre-B cell stage^24^. To investigate class IA PI3K function in early B cells, we expressed constitutively active versions of p110α (myr-p110α) or AKT (myr-AKT) in a bone marrow (bm)-derived wild type (wt) pre-B cell line (Fig. S1a). GFP-negative and empty vector-transduced cells behaved comparably. For the sake of simplicity only the latter will be shown in further experiments (Fig. 1a). As expected, phosphorylated AKT (pAKT) indicative of activation was increased in pre-B cells expressing myr-p110α or myr-AKT (Fig. 1a). Moreover, we found that Pax5 and CD19 expression was elevated in these cells (Fig. 1a and Fig. S1b and data not shown). Notably, neither myr-AKT nor myr-p110α did lead to IL-7 independent cell growth (data not shown). To confirm the finding that PI3K signaling induces Pax5 expression, we treated the bm-derived wt pre-B cell line with the PI3K-inhibitor LY294002 at a concentration ensuring a robust decline in AKT activity while not interfering with cell viability at the time of analysis (Fig. 1b and Fig. S1c). The results show that inhibition of PI3K signaling interferes with Pax5 expression. Since Pax5 activates SLP-65 expression^4^ we tested whether class IA PI3K also regulates SLP-65. Indeed, treatment of cells from the bm-derived wt pre-B cell culture with the PI3K-inhibitor LY294002 led to downregulation of both Pax5 and SLP-65 protein expression (Fig. 1b). Consistent with the negative effect of activated PI3K, FoxO1-levels increased upon LY294002 application (Fig. 1b).

To test whether class IA PI3K-dependent regulation of Pax5 also acts in the presence of continuous pre-BCR signaling as well as for later B cell developmental stages, we utilized a SLP-65-deficient pre-B cell line (Fig. 1c) and primary mature B cells (Fig. 1d, Fig. S1d), respectively. Expression of Pax5 declined in both cell types upon inhibition of PI3K signaling on protein (Fig. 1c,d) and transcript level (Fig. 1e,f). Additionally, PI3K-mediated activation of Pax5 expression was also detected in pre-B and mature B cells of human origin (Fig. S1f,g). Together, class IA PI3K signaling activates Pax5 expression irrespective of the species and of the B cell developmental stage.

### PI3K requires the pre-BCR but not the IL-7R

**Figure 2:**
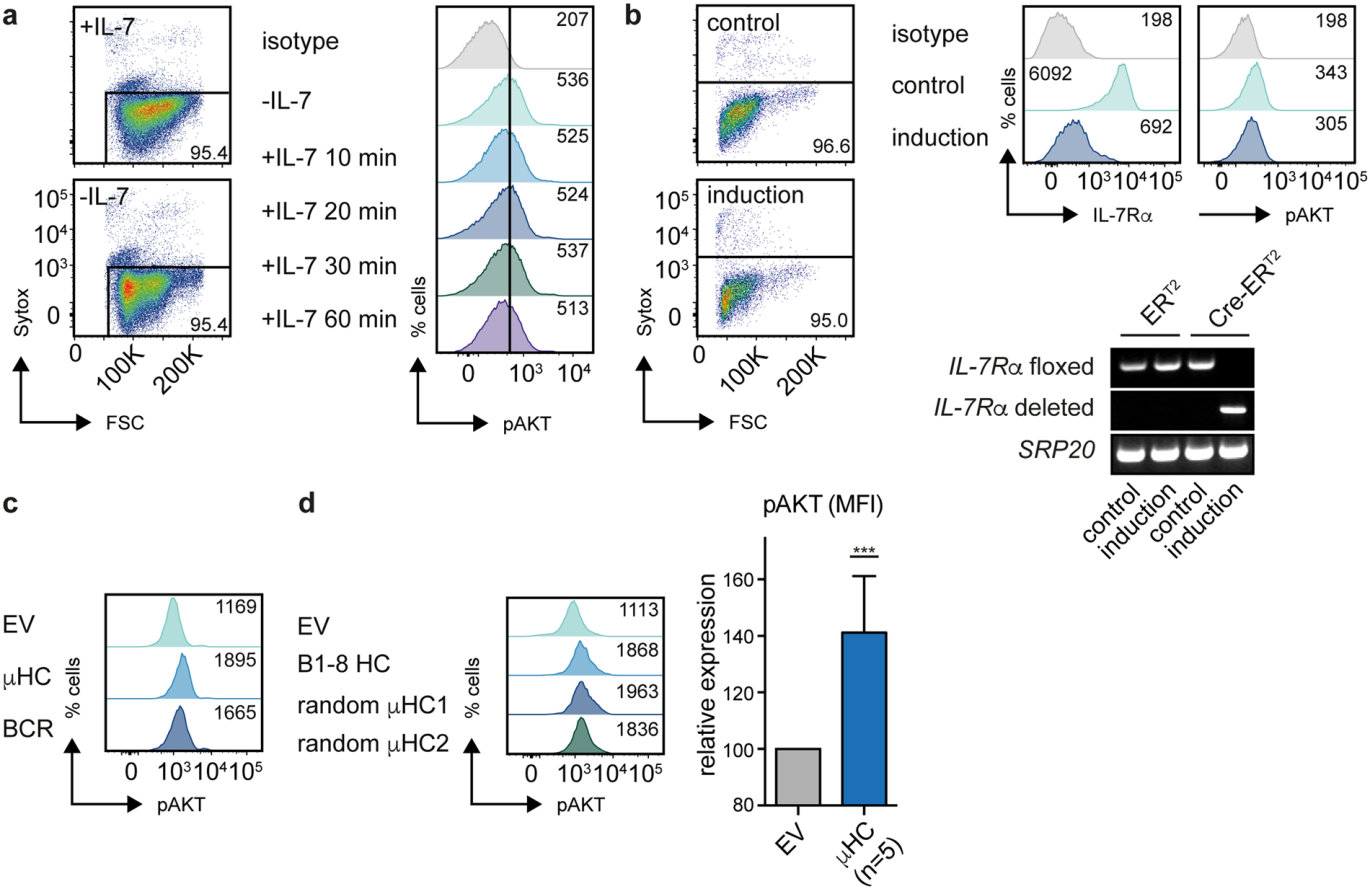
Pre-BCR activates PI3K signaling, whereas IL-7R does not. (**a**) Cells from a bm-derived wt pre-B cell line were cultured overnight in presence or absence of IL-7. Viability of the cells was assessed by Sytox staining (left panel). Levels of pAKT were measured by FACS at the indicated time points following addition of IL-7 (10 ng/ml) (right panel). Numbers in the dot plots indicate the percentages, numbers in the histogram plots indicate the MFI. (**b**) Cells from an *IL-7R*α^fl/fl^ bm-derived pre-B cell line were transduced with a 4-hydroxytamoxifen (4-OHT)-inducible form of Cre-recombinase (Cre-ER^T2^) or ER^T2^ as EV and treated with 4-OHT (induction) or EtOH (control) for 48 h. Viability of the cells was assessed by Sytox staining (left). Surface levels of IL-7Rα as well as intracellular levels of pAKT were determined by FACS analysis (right, top). Additionally, Cre-mediated deletion was confirmed by PCR using specific primers detecting the floxed or deleted *IL-7R*α allele (right, bottom). PCR for SRP20 was used as loading control. For original full-length gel pictures see Figure S2b. (**c**) Cells from a Rag2-deficient pro-B cell line were retrovirally transduced with μHC (B1-8 HC), or μHC + λLC (BCR) and levels of pAKT were measured at day 2 upon transduction by FACS. Numbers in the histogram plots indicate the MFI. (**d**) Indicated μHCs were introduced into cells from a Rag2-deficient pro-B cell line as in (**c**) and analyzed at day 1 post transduction for levels of pAKT by FACS (left). Numbers in the histogram plots indicate the MFI. Indicated μHCs were expressed at least 3 times and the average MFI of pAKT FACS-analysis over all experiments is shown (right). Statistical significance was calculated using the t-Test. Data shown in Fig. 2a,b are representative of at least 3 independent experiments.

Besides pre-BCR, IL-7R-derived signals play important roles during early B cell development^7^. To test whether IL-7R activates PI3K signaling, we incubated bm-derived wt pre-B cells overnight in the absence of IL-7, treated the cells with IL-7 and after different incubation periods within 60 min we determined AKT phosphorylation. The results show that pAKT was not increased after IL-7 treatment at any time point tested (Fig. 2a). To provide additional evidence for the dispensable role of IL-7 in PI3K activation, we used a bm-derived pre-B cell line carrying loxP-flanked *IL-7R*α alleles allowing Cre-mediated deletion of *IL-7Rα* and thus abrogating IL-7R signaling^28^. Indeed, deletion of *IL-7R*α showed no effect on the pAKT-levels (Fig. 2b and Fig. S2a). Moreover, absence of IL-7 further increased expression of CD19 by myr-AKT and myr-p110α (Fig. S1b). Since pre-BCR expressing cells can be further divided into (IL-7 responsive) large pre-B cells and (IL-7-non-responsive) small pre-B cells, we tested whether *IL-7Rα* deletion in IL-7-responsive large pre-B cells can affect the phosphorylation level of AKT. Indeed, even with this experimental setup, AKT phosphorylation was not changed after *IL-7Rα* deletion (Fig. S3a). These data demonstrate that IL-7R is unlikely to be essential for PI3K activation in early B cell development.

To test whether pre-BCR signaling activates PI3K signaling, we reconstituted the pre-BCR in a Rag2-deficient pro-B cell line. Since Rag2-deficient cells express an endogenous surrogate LC, introduction of μHC results in pre-BCR expression in these cells. The data show that μHC leads to increased AKT phosphorylation suggesting that both the pre-BCR and the BCR activate PI3K signaling (Fig. 2c). To confirm these data, we used additional μHCs including 2 randomly cloned μHCs from the spleen of wt mice (Fig. 2d). Interestingly, we found that pre-BCR-induced AKT phosphorylation is detectable within 24–72 h (Fig. 2d) and that AKT phosphorylation is decreased after this time period (Fig. S3b), suggesting that activation of PI3K signaling by the pre-BCR is a regulated process. In addition to the increase in pAKT, μHC expression in Rag2-deficient pro-B cells also resulted in elevated Pax5 levels (Fig. S3c). Together, our data suggest that signaling by the pre-BCR, and not by IL-7R, is required for stimulation of PI3K activity in early B cell development.

### PI3K signaling regulates plasticity of developing B cells

**Figure 3:**
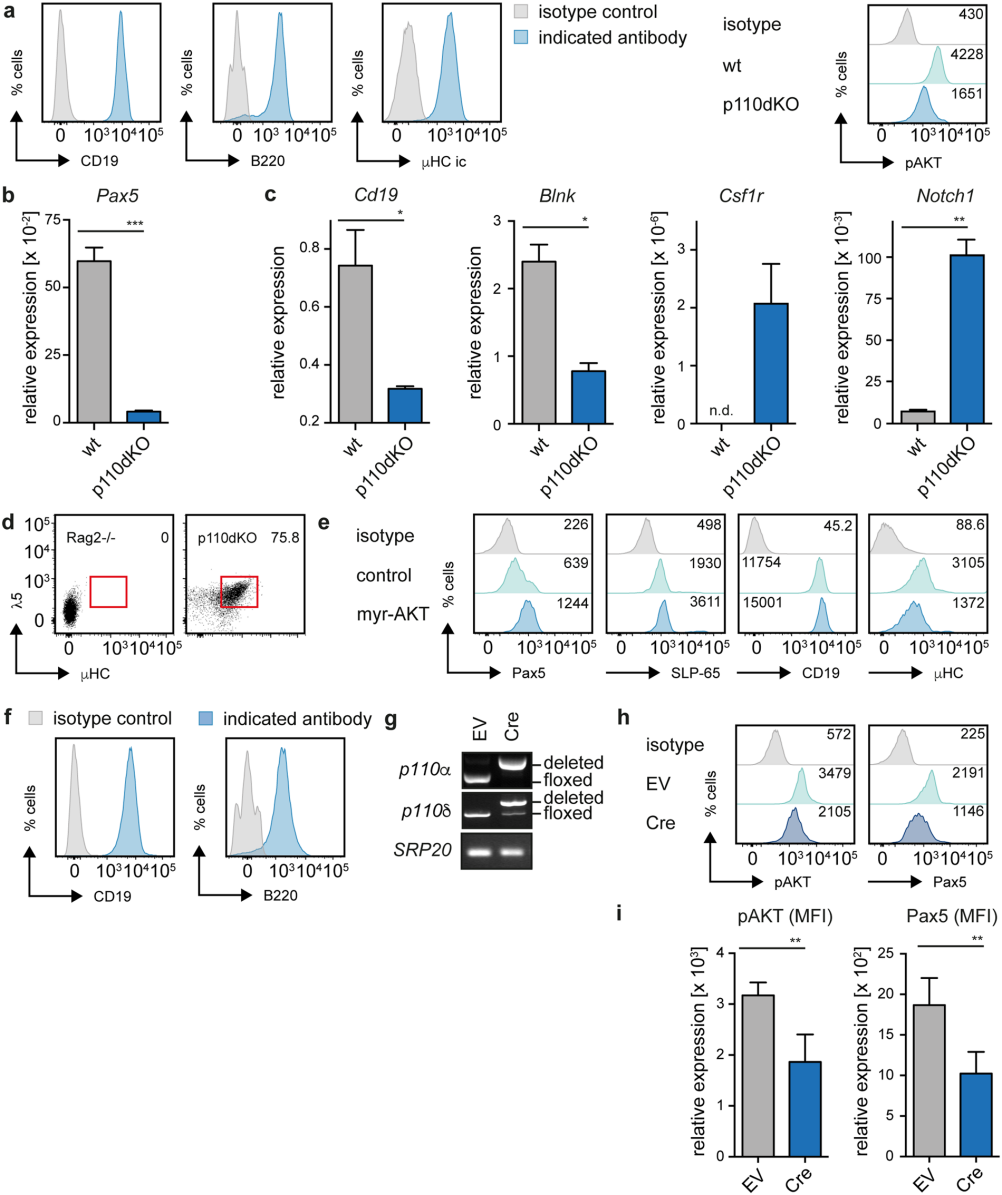
PI3K-deficient cells are blocked in differentiation and show imperfect B lineage commitment. (**a**) p110dKO cells were stained for CD19, B220 (both surface) and μHC (ic, intracellular) or the respective isotype control and analyzed by FACS (left panel). Cells from bm-derived wt and p110dKO pre-B cell lines were analyzed for pAKT levels by intracellular FACS (right panel). (**b**,**c**) Total RNA was isolated from bm-derived wt and p110dKO pre-B cell cultures to analyze *Pax5*, *Cd19*, *Blnk*, *Csf1r* and *Notch1* mRNA levels with specific primers by qRT-PCR using the SYBR-Green detection method. Results are shown as mean ± SD of 2 independent analyses, run as duplicates. Statistical significance was calculated using the Mann-Whitney Test or the t-Test. (**d**) FACS analysis of p110dKO cells for surface expression of the pre-BCR components μHC and λ5. Cells from a Rag2-deficient B cell culture served as negative control. Numbers indicate percentages of cells in the respective region. (**e**) p110dKO cells were retrovirally transduced with myr-AKT or the EV. Expression of Pax5, SLP-65 (both intracellular), CD19 and μHC (both surface) was analyzed by FACS at day 2 after transduction and compared with expression in untransduced bystander cells (control). Data are representative of at least 3 independent experiments. n. d.: not detectable. (**f**) An IL-7 dependent pre-B cell line was established from bm of *p110α*^fl/fl^/*p110δ*^fl/fl^ mice. The respective cells were characterized by surface staining for CD19 and B220, or the respective isotype control and analyzed by flow cytometry. (**g**) Cells described in Fig. 3f were retrovirally transduced with a Cre-encoding expression vector or EV, respectively. Cre-mediated deletion of *p110α* and *δ* was confirmed by PCR using specific primers detecting floxed or deleted alleles. PCR for *SRP20* served as loading control. For original full-length gel pictures see Figure S4c. (**h**) Cells described in Fig. 3f were retrovirally transduced with a Cre-encoding expression vector or EV, respectively, and analyzed by intracellular FACS for pAKT and Pax5 expression. Numbers in the histogram plots indicate the MFI, depicted data are representative of at least 4 independent experiments. (**i**) Average MFIs of pAKT and Pax5 following Cre-mediated *p110α* and *p110δ* deletion. Results are shown as mean ± SD of 4 independent experiments. Statistical significance was calculated using the t-Test.

Our data show that class IA PI3K signaling is mediated by the pre-BCR and that PI3K activates Pax5 and SLP-65 expression, which are important for pre-B cell differentiation. This novel link suggests that PI3K signaling activates pre-B cell differentiation. To test the role of PI3K signaling in differentiation^24^, we used bone marrow cells from a *p110α*^−/−^/*δ*^D910A^ mouse to generate an IL-7-dependent pre-B cell line (Fig. 3a, hereafter referred to as p110dKO cells, Fig. S4a). Signaling through class IA PI3K was affected in p110dKO cells as shown by reduced levels of pAKT (Fig. 3a). In accordance with our previous findings, p110dKO cells showed reduced transcription of *Pax5* (Fig. 3b) and of its target genes *Cd19* and *Blnk* (encoding SLP-65) (Fig. 3c).

Since signaling through SLP-65 regulates pre-BCR expression, we tested whether PI3K-deficient p110dKO cells showed defective pre-BCR down-regulation^10,11^. Indeed, we detected increased pre-BCR expression on the surface of p110dKO cells (Fig. 3d). To confirm the block in pre-BCR signaling in p110dKO cells, we tested whether expression of myr-AKT was able to restore expression and function of SLP-65. Indeed, myr-AKT led to increased expression of Pax5, CD19 and SLP-65 as well as down-regulation of surface pre-BCR expression (Fig. 3e). To provide additional evidence for the importance of PI3K signaling in activation of Pax5 expression, we established an IL-7-dependent pre-B cell line from the bone marrow of mice carrying loxP-flanked alleles for *p110α* and *p110δ*, the main catalytic p110 subunits acting in B cells^24^ (Fig. 3f and Fig. S4b). Deletion of *p110α/p110δ* by retroviral transduction of Cre expression vector resulted in decreased pAKT and Pax5 expression (Fig. 3g–i) suggesting that PI3K activity is important for maintaining the transcriptional program of developing B cells.

### PI3K deficiency results in unstable B cell commitment

**Figure 4:**
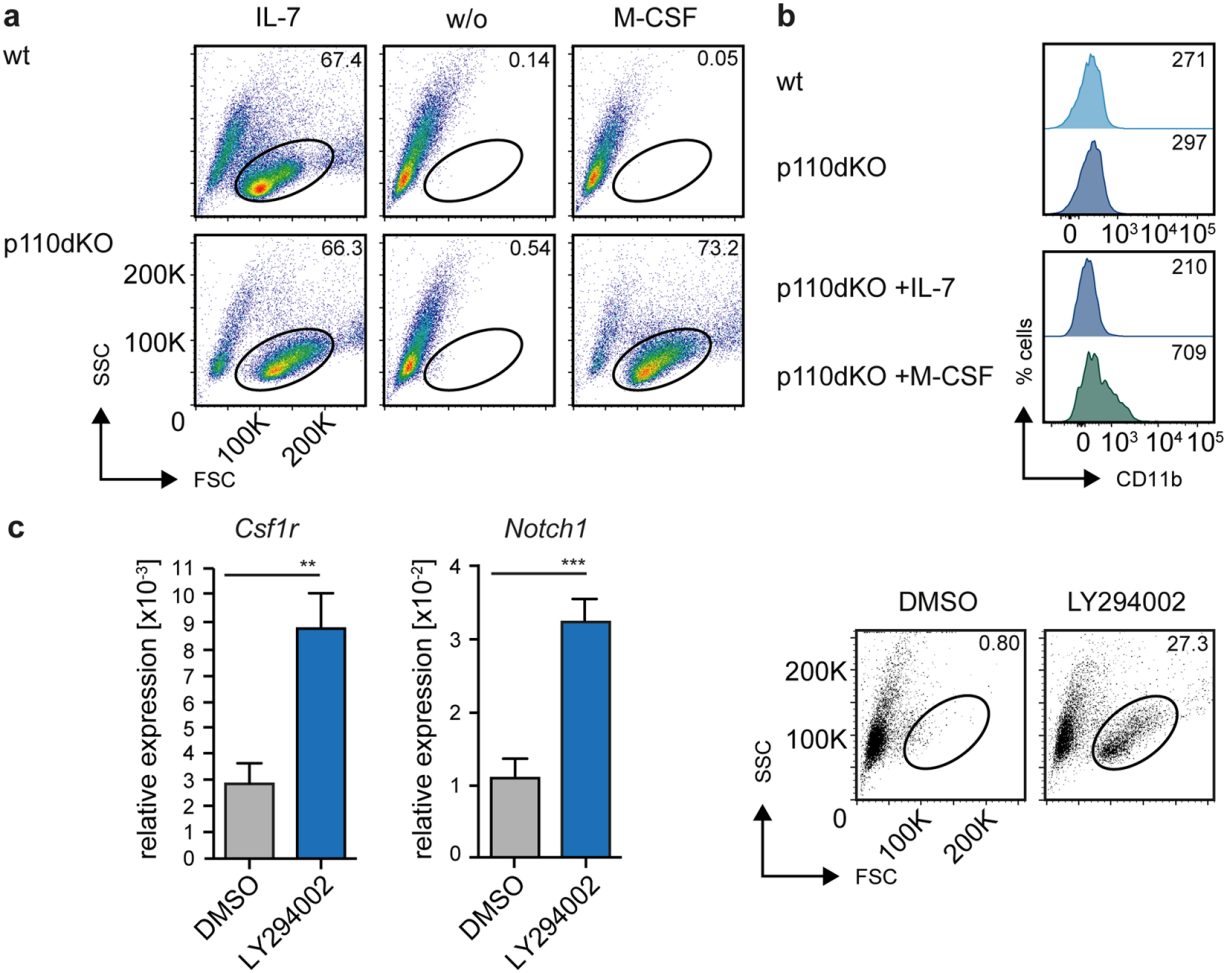
PI3K signaling regulates B cell commitment and plasticity. (**a**) Cells from bm-derived wt and p110dKO pre-B cell lines were cultured either in the presence of IL-7, M-CSF, or without cytokines and analyzed by flow cytometry. Numbers indicate percentages of cells in the respective regions at day 7 after beginning of treatment. (Data are representative of at least 3 independent experiments). (**b**) FACS-analysis of CD11b expression of measured in cells from bm-derived wt and p110dKO pre-B cell lines cultured in the presence of IL-7 (upper panel). p110dKO cells were cultured in IL-7 or M-CSF supplemented medium and CD11b expression was assessed by FACS analysis at day 8 after beginning of treatment (lower panel). (**c**) Cells from a bm-derived SLP-65-deficient pre-B cell line were treated with LY294002 (30 μM) or solvent (DMSO) for 16 h and subsequently cultured in medium supplemented with M-CSF. *Csf1r* and *Notch1* mRNA levels were determined with specific primers by qRT-PCR using the SYBR-Green detection method (left panel). Results are shown as mean ± SD of 2 independent analyses, run as duplicates. Statistical significance was calculated using the Mann-Whitney Test or the t-Test. Cells from the respective culture conditions were further analyzed by flow cytometry (right panel).

Given the importance of Pax5 for B-cell lineage specific gene expression, the link between class IA PI3K and Pax5 suggested that PI3K-deficient p110dKO pre-B cells show defective B-lineage gene expression because of reduced Pax5 activation. In fact, we detected transcripts of *Csf1r* and *Notch1* (Fig. 3c). *Csf1r* encodes the colony stimulating factor 1 receptor (CSF1R), also known as macrophage colony-stimulating factor receptor (M-CSFR), which is usually expressed on macrophages and is repressed by Pax5 in B cells. Based on the presence of *Csf1r* transcripts in p110dKO cells we tested whether p110dKO cells were able to respond to macrophage-colony stimulating factor M-CSF, the ligand of CSF1R and the factor, essential for survival, growth and differentiation of macrophages^29^. In contrast to wt cells, p110dKO pre-B cells survived in medium containing M-CSF (Fig. 4a and Fig. S5a) and expressed the myeloid specific surface marker CD11b (Mac-1) (Fig. 4b) suggesting that failure to suppress CSF1R expression in p110dKO cells allows survival under myeloid culture conditions. We further confirmed that p110dKO cells surviving in M-CSF-supplemented culture conditions were of B cell lineage origin since both the original IL-7-dependent cell line and the M-CSF-dependent cell line showed the same VDJ recombination pattern (Fig. S5b). Additionally, we tested *in vivo* the potential of p110dKO to enter the T-cell lineage by injecting p110dKO cells into Rag2/common γ chain double deficient mice. Interestingly, we found that mice injected with p110dKO developed a thymus-like structure in which we could detect CD4^−^, CD8^−^ as well as CD4^+^/CD8^+^ cells (Fig. S5c,d).

Since the analysis of p110dKO cells suggested a role of PI3K in B cell identity, we investigated whether blocking PI3K activity can induce lineage plasticity of PI3K-sufficient cells. Indeed, we detected elevated levels of *Csf1r* and *Notch1* transcripts in cells from a bm-derived SLP-65-deficient pre-B cell line treated with LY294002 (Fig. 4c). To test whether PI3K-inhibitor treatment enabled these cells to survive under non-B cell conditions, LY294002-treated cells were cultured in medium supplemented with M-CSF. In fact, LY294002-treated pre-B cells survived under myeloid conditions (Fig. 4c), indicating that PI3K-sufficient pre-B cells become responsive to a myeloid specific growth factor after inhibition of PI3K.

Together, these data suggest that PI3K-deficiency blocks B cell development at the pre-B cell stage due to the inability of PI3K-deficient pre-B cells to induce Pax5 and thus the differentiation program for developing B cells.

### FoxO1 is involved in Pax5 regulation

**Figure 5:**
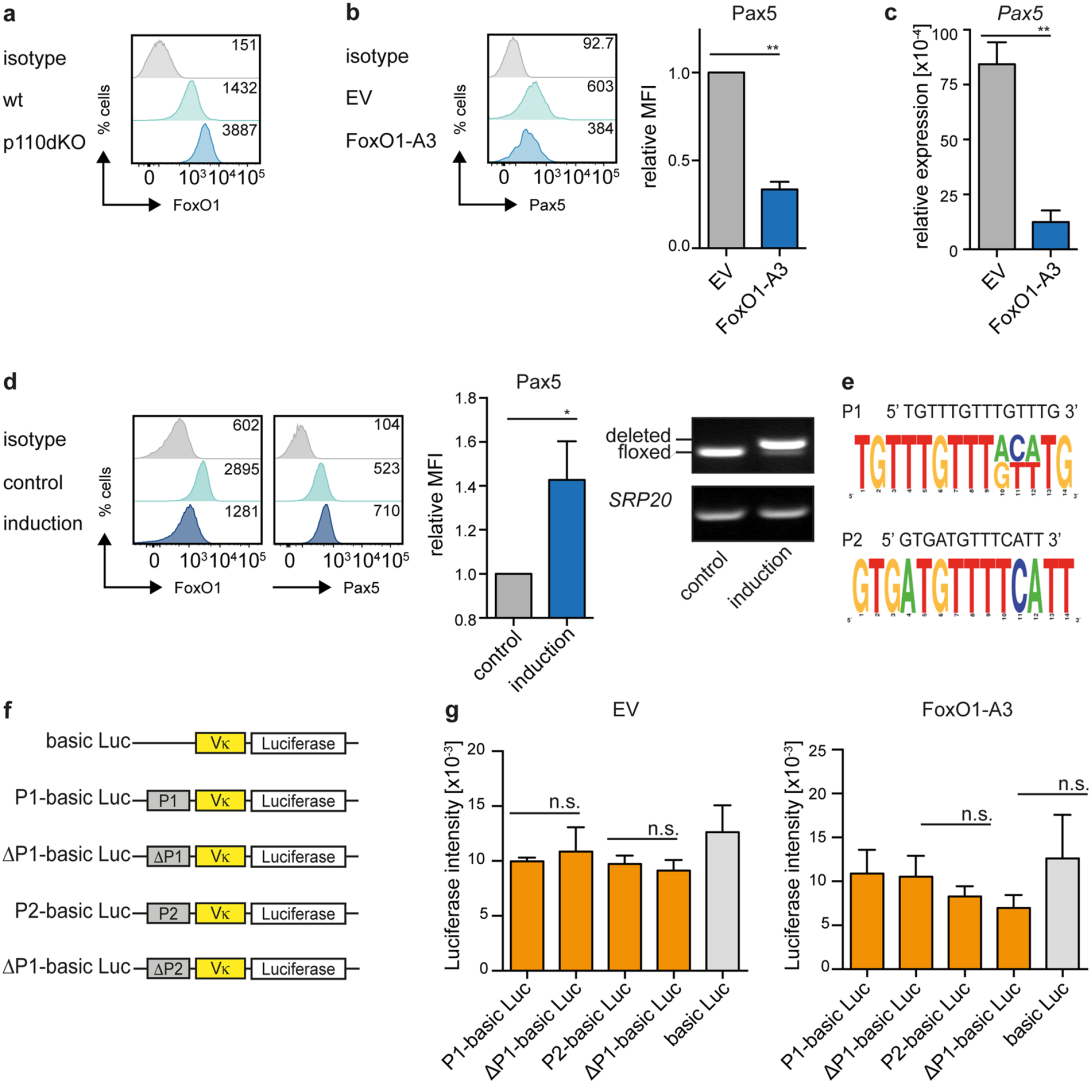
PI3K regulates Pax5 via FoxO1. (**a**) Cells from bm-derived wt and p110dKO pre-B cell lines were analyzed by intracellular FACS for FoxO1. (**b**) Cells from a bm-derived wt pre-B cell line were transduced with a constitutively active form of FoxO1 (FoxO1-A3) or an EV control. Expression of Pax5 was assessed by intracellular FACS at day 2 after transduction in at least 3 independent experiments. Statistical significance was calculated using the t-Test. (**c**) Total RNA of cells from Fig. 5b was isolated to analyze transcript levels of *Pax5*. *Hprt* and *Pax5* mRNA-levels were detected by qRT-PCR using the SYBR-Green detection method. Results are shown as mean ± SD of 2 independent experiments, run as duplicates. Statistical significance was calculated using the t-Test. (**d**) Cells from a *FoxO1*^fl/fl^ bm-derived pre-B cell line were transduced with Cre-ER^T2^ or -ER^T2^. 4-OHT was applied to activate the Cre-ER^T2^ (induction), treatment with EtOH served as control. Expression levels of FoxO1 and Pax5 were analyzed by intracellular flow cytometry (left panel). The MFI of Pax5 upon deletion of FoxO1 was quantified (bar diagram). Statistical significance was calculated using the t-test. Deletion of the floxed *FoxO1* allele was confirmed by PCR, *SRP20* served as loading control (right). For original full-length gel pictures see Fig. S2d. Depicted data are representative of at least 3 independent experiments. (**e**) Sequences of two FoxO1-binding motifs identified by the Encode Genome Project. Site 1 (P1) is located in intron 5, site 2 (P2) in intron 9 of the murine *Pax5* gene. P1 and P2 are highly conserved between man and mouse. Graphs were prepared using the WebLogo software. (**f**) Schematic overview of the luciferase expression vector harboring a Vκ21 promoter and a 1 kb fragment containing or lacking the potential FoxO1 binding sites shown in Fig. 5e. (**g**) WEHI cells were electroporated to introduce the empty vector (Vκ + Luciferase) or the indicated constructs containing or lacking the potential FoxO1 binding sites. Expression of luciferase was equalized to the rLUC(Renilla) expression in each sample and WEHI cells were cotransfected either with EV (CMV-EV) or with a vector encoding FoxO1-A3 (CMV-FoxO1-A3). Data are representative of 3 (EV) and 4 (FoxO1-A3) independent experiments and luciferase expression was determined using the Dual-Luciferase Reporter Assay System (Promega).

To characterize the molecular mechanism underlying the PI3K-mediated activation of Pax5 we investigated the role of FoxO1, which is known to be a highly conserved downstream target of PI3K/AKT signaling and is essential for *Ig* gene recombination^30,31^. Signaling through class IA PI3K/AKT results in proteasomal degradation of FoxO1, whereas in the absence of PI3K-activity, FoxO1 protein is stabilized^32^. In fact, we detected elevated levels of FoxO1 in p110dKO pre-B cells (Fig. 5a). To test whether FoxO1 was involved in the PI3K-dependent regulation of Pax5, we introduced a constitutively active form of FoxO1 (FoxO1-A3) or an empty control vector (EV) into cells from a bm-derived wt pre-B cell line and found that FoxO1-A3 led to reduced Pax5 expression (Fig. 5b,c). Furthermore, inducible Cre-mediated deletion of loxP-flanked *FoxO1* in a bm-derived pre-B cell line resulted in higher expression of Pax5 (Fig. 5d and Fig. S2c).

Since FoxO1 has been shown to directly bind to the *Pax5* gene^33^, we investigated whether FoxO1 represses *Pax5* transcription through potential FoxO1-binding sites in the *Pax5* gene. Two highly conserved, potential FoxO1-binding sites within the *Pax5* gene locus were identified (Fig. 5e) and used for the generation of luciferase expression vectors. These vectors carry, upstream of a basal Vκ-promotor, 1 kb-fragments from the *Pax5* gene containing or lacking the potential FoxO1-binding sites (Fig. 5f). However, no significant effects of these conserved FoxO1-binding sites were observed on luciferase expression (Fig. 5g). These results suggest that FoxO1 might regulate Pax5 through an indirect mechanism.

### FoxO1 represses Pax5 by induction of Irf4

**Figure 6:**
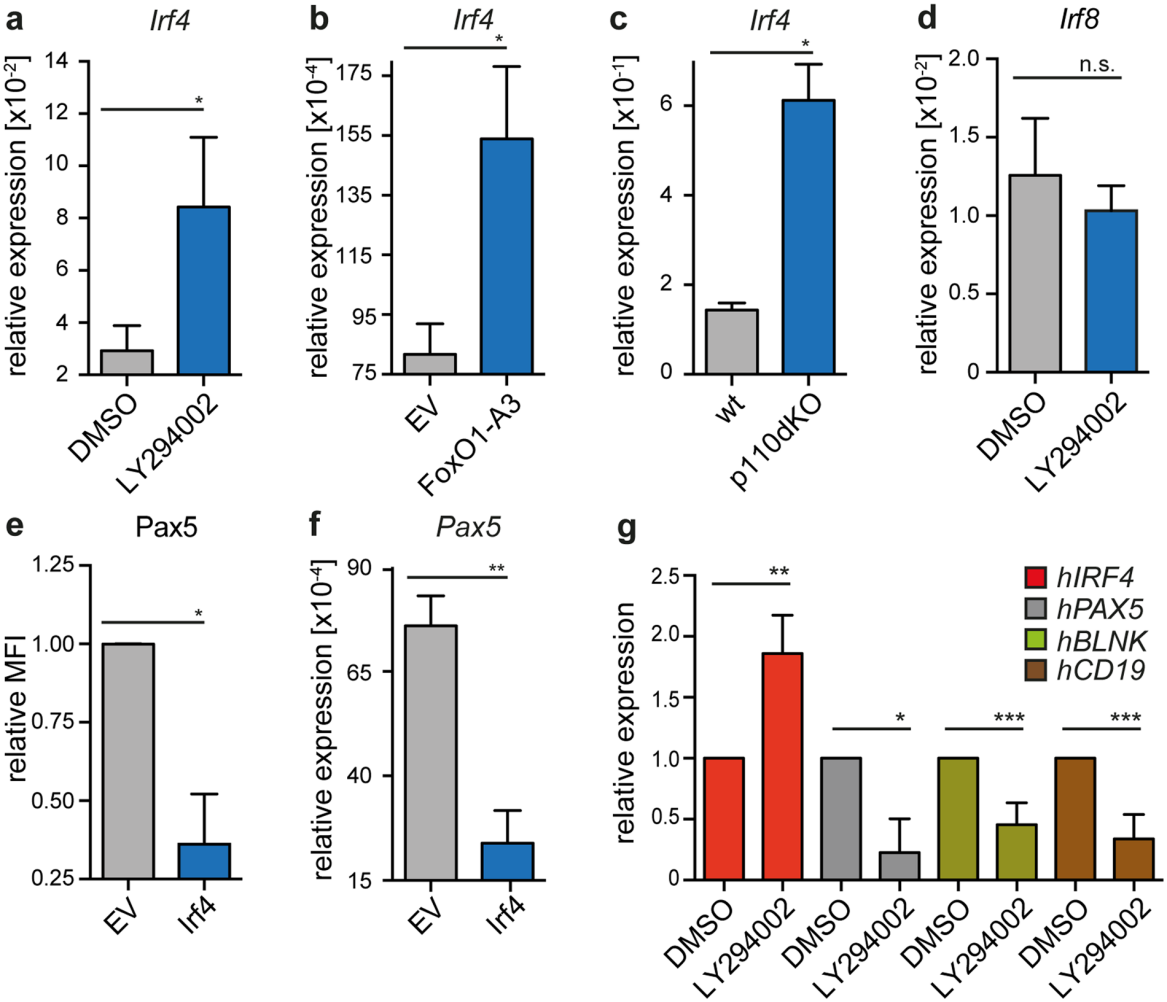
FoxO1 induces Irf4 to repress Pax5. (**a**) Total RNA from a SLP-65-deficient pre-B cell line, treated with LY294002 or DMSO for 12 h, was isolated. *Hprt* and *Irf4* mRNA-levels were detected with specific primers by qRT-PCR using the SYBR-Green detection method (n = 5). (**b**) Cells from a bm-derived wt pre-B cell line were transduced with FoxO1-A3 or EV and at day 1 post transduction total RNA was isolated. Expression of *Hprt* and *Irf4* mRNA-levels were detected by qRT-PCR using a SYBR-Green detection method (n = 3). (**c**) Total RNA from p110dKO and bm-derived wt pre-B cell lines was analyzed for *Hprt* and *Irf4* transcript levels by qRT-PCR using the SYBR-Green detection method (n = 3). (**d**) *Hprt* and *Irf8* mRNA-levels were detected in RNA from Fig. 6a with specific primers by qRT-PCR using the SYBR-Green detection method (n = 5). (**e**) Similar to FoxO1-A3 in Fig. 5b, cells from a bm-derived wt pre-B cell line were transduced with Irf4 or EV and Pax5 expression was analyzed at day 2 after transduction by intracellular FACS. (**f**) Total RNA of cells from Fig. 6e was isolated to analyze transcript levels of *Pax5*. *Hprt* and *Pax5* mRNA-levels were detected by qRT-PCR using the SYBR-Green detection method (n = 3). (**g**) Human mature B cells from peripheral blood were isolated, treated with LY294002 and analyzed for *IRF4*, *PAX5*, *BLNK* and *CD19* transcripts (n = 3). Results are shown as mean ± SD, run as duplicates. Data are representative of 2 (**c**) or at least 3 (**a,b**, **d–g**) independent experiments. Statistical significance was calculated using the t-Test.

A search for transcription factors that are activated by FoxO1 revealed interferon regulatory factor 4 (Irf4) as a potential candidate for Pax5 regulation. In fact, the *Irf4*-promoter contains specific sites for FoxO1 binding and subsequent activation of *Irf4* expression^34^. Furthermore, Irf4 was shown to bind the *Pax5* gene within a recently identified enhancer region^35^. To investigate the role of Irf4 in PI3K-dependent regulation of Pax5 expression, we confirmed that *Irf4* is induced upon treatment of SLP-65-deficient pre-B cells with the PI3K inhibitor LY294002 (Fig. 6a) or after introduction of FoxO1-A3 into a bm-derived wt pre-B cell line (Fig. 6b). In line with these findings, p110dKO pre-B cells showed elevated levels of *Irf4* transcripts (Fig. 6c). Notably, *Irf8*, which is structurally highly related to Irf4, was not regulated by PI3K (Fig. 6d) and did not suppress Pax5 expression (Fig. S6). To directly test whether Irf4 links FoxO1 to the regulation of *Pax5* gene transcription, we transduced cells from a wt pre-B cell line with Irf4 expression vectors and found that Pax5 protein and transcript amounts were reduced after Irf4 expression (Fig. 6e,f). Additionally, we tested whether the inverse regulation of *Irf4* and *Pax5* can also be detected in human peripheral B cells. Indeed, PI3K-inhibition of freshly isolated mature B cells from peripheral blood led to increased *IRF4* and lower *PAX5*, *CD19* as well as *BLNK* gene transcription (Fig. 6g). Together, these data clearly suggest that class IA PI3K induces Pax5 by inhibiting FoxO1 and thereby relieving Irf4-repression of Pax5.

### PI3K-responsive element (PIRE) in *Pax5* gene

**Figure 7:**
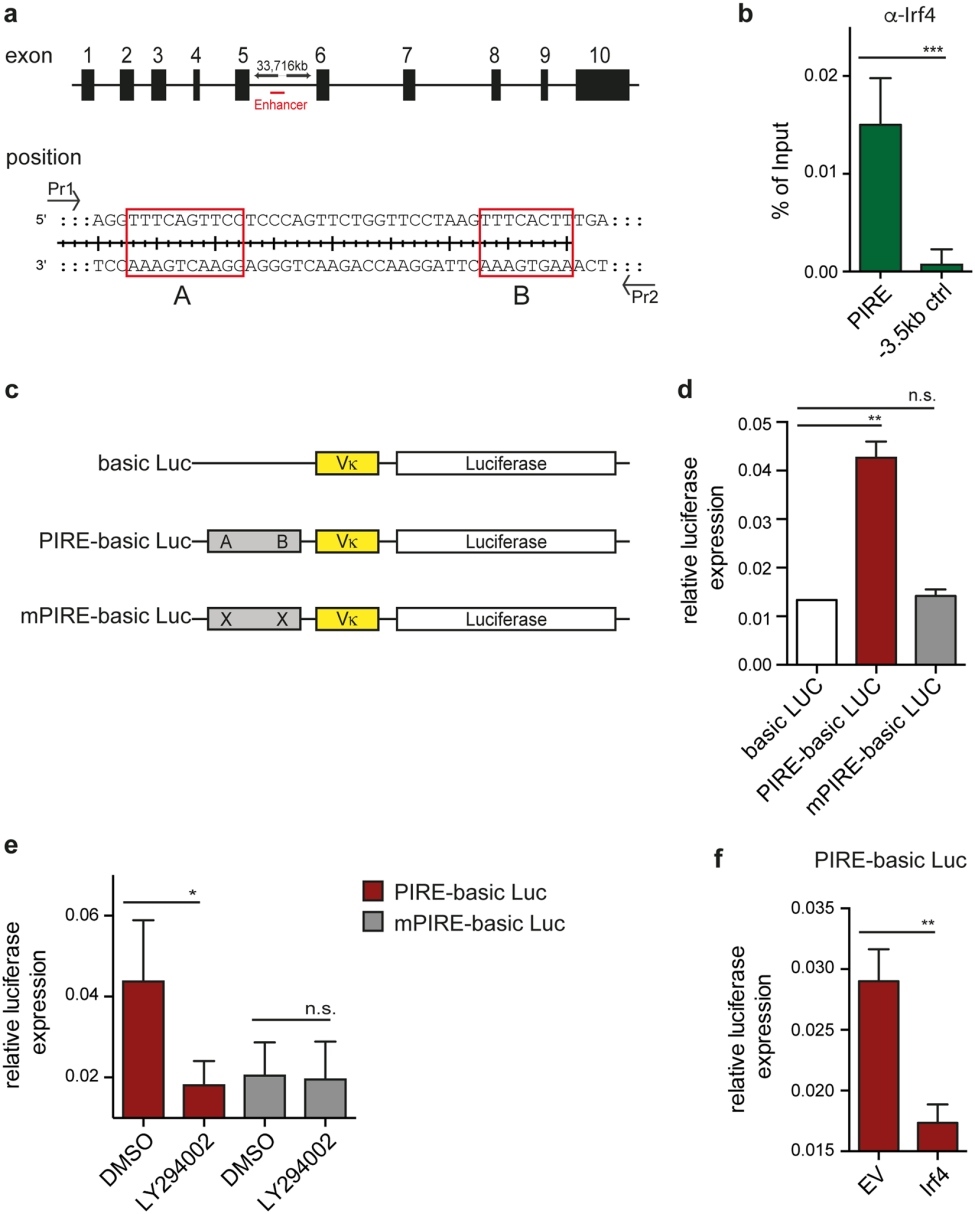
Irf4 directly represses Pax5 expression. (**a**) Schematic overview showing the location of the Pax5 enhancer region within the Pax5 gene locus. (**b**) Irf-4 ChIP from cells of a SLP-65-deficient pre-B cell line. Amplified by qPCR was the enhancer region of Pax5 within exon 5 containing Irf4-binding sites (A and B). As control, amplification of a region 3.5 kb upstream of Pax5 enhancer was chosen. Both samples were normalized to mock control. (**c**) Schematic overview of the luciferase expression vector harboring a Vκ promoter and the respective Pax5 enhancer region containing or lacking the Irf4 binding sites. (**d–f**) WEHI cells were electroporated to introduce the empty vector (Vκ + Luciferase) or the indicated constructs containing or lacking the Irf4 binding sites. Expression of luciferase was equalized to the rLUC (Renilla) expression in each sample of co-transfected WEHI cells. In (**e**), WEHI cells were treated for 16 h with LY294002 upon electroporation and in (**f**), WEHI cells were co-transfected with either EV or with a vector encoding Irf4. Data are representative of at least 3 independent experiments and luciferase expression was determined using the Dual-Luciferase Reporter Assay System.

To further characterize the molecular link between PI3K, FoxO1, Irf4 and Pax5, we first confirmed the binding of Irf4 to the previously described *Pax5* enhancer region^35^ containing two Irf binding motifs (Fig. 7a,b). To test whether class IA PI3K and Irf4 influence the activity of this enhancer region, we generated luciferase expression vectors using *Pax5*-derived DNA sequences containing or lacking the Irf4-binding sites (Fig. 7c, PIRE-basic Luc and mPIRE-basic Luc, respectively). Consistent with the described enhancer function of this *Pax5* gene region, we found elevated luciferase expression in presence of the Irf4-binding motifs (Fig. 7d). Notably, inactivating these Irf4-binding motifs (mPIRE-basic Luc) abolished this enhancer function (Fig. 7d). To test whether class IA PI3K activity affects the enhancer function, we treated cells expressing PIRE- or mPIRE-basic Luc with LY294002. Interestingly, luciferase expression declined upon PI3K inhibition in PIRE- but not mPIRE-expressing cells (Fig. 7e), indicating that the activity of class IA PI3K critically influences *Pax5* enhancer function. Since our data, presented so far, suggested that class IA PI3K regulates Pax5 via Irf4, we tested next whether increased expression of Irf4 counteracts luciferase expression in PIRE-expressing cells. Indeed, enforced expression of Irf4 repressed PIRE-induced luciferase expression confirming the repressive effect of Irf4 on the fragment containing PIRE (Fig. 7f). Together, these results suggest that class IA PI3K controls B cell development by induction of *Pax5* gene expression through the activation of the *Pax5* enhancer region.

## Discussion

The PI3K signaling pathway is essential for the development of pre-B cells and for the maintenance of mature B cells^36^. In this study, we show that the crucial role of PI3K in B cell differentiation is mediated by activating the expression of Pax5 through a mechanism involving FoxO1 and Irf4. Accordingly, the combined deficiency of p110α and p110δ in p110dKO pre-B cells results in severe alteration in the expression of Pax5 and Pax5-regulated genes, which are required for pre-B cell development. Our data point to FoxO1 and Irf4 as an important molecular link between PI3K activity and Pax5 expression. Although it is conceivable that additional elements might be involved in the PI3K-mediated induction of Pax5, available data support the role of Irf4 as a potential link between PI3K signaling and *Pax5* gene expression. For instance, it has been shown that Irf4-levels are elevated in p110δ-deficient mature B cells and that inhibition of p110δ induces *Irf4* gene transcription^37^. The finding that FoxO1 directly binds to the *Irf4* promoter and induces the expression of Irf4 suggests that PI3K activity represses Irf4 by activating the degradation of FoxO1^34^. This is in agreement with our results showing that class IA PI3K signaling represses Irf4 thereby activating Pax5. Since the ability of Irf4 to function as a transcriptional repressor is well established^38^. Our results suggest that Irf4 represses *Pax5* gene transcription by directly binding to the enhancer region of the *Pax5* gene. Notably, a previous study showed that a large fragment of the *Pax5* enhancer containing the Irf4 binding site in addition to other transcription factors activated the transcription of *Pax5* in a transient transfection assay^35^. Together with our data, this suggests that the outcome of Irf4 on Pax5 expression may depend on the activity of additional transcription factors that also bind to the *Pax5* enhancer. Consequently, modulating the activity of the involved transcription factors during B cell development determines the effect of Irf4 on Pax5 expression. Moreover, the identification of FoxO1 regulating Irf4 provides a molecular rationale as to how SLP-65-activation results in Irf4-induction and thereby in initiation of κLC-expression^39^. Previous findings showed furthermore that Pax5 induces SLP-65, which downregulates PI3K and activates FoxO1 and Irf4^4,9,39^, the FoxO1/Irf4-mediated repression of Pax5 interferes with SLP-65 expression, thereby regulating FoxO1/Irf4 activation. Thus, SLP-65 amounts control the balance between Pax5 and FoxO1/Irf4 by regulating PI3K signaling activity and therefore, characterizing the exact roles of these transcription factors is essential for understanding pre-B cell commitment and differentiation.

The essential role of PI3K signaling in the initiation of pre-B cell differentiation is supported by studies that directly interfere with PI3K function in developing B cells. For instance, combined deletion of BCAP and CD19 leads to severely impaired AKT activation upon BCR stimulation and the number of early pre-B cells is strongly increased in these mice^16^. In contrast, the number of late pre-B cells is markedly reduced, suggesting that, in the absence of both BCAP and CD19, the impaired activation of PI3K results in defective differentiation of large pre-B cells to the small pre-B cell stage^16^. This observation is in complete agreement with our finding that class IA PI3K-derived signals are required for inducing the expression of Pax5 and the downstream target SLP-65. In fact, p110dKO pre-B cells deficient for PI3K signaling, express reduced amounts of SLP-65 and high amounts of the pre-BCR on their cell surface. This suggests that the residual SLP-65 expression in p110dKO pre-B cells is not sufficient to activate SLP-65-dependent signaling that leads to pre-BCR down-regulation and further differentiation^10^. Therefore, we propose that the amounts of Pax5 and SLP-65 at the onset of pre-BCR expression are insufficient to drive the differentiation processes. Increased PI3K signaling mediated by pre-BCR function results in elevated amounts of Pax5 and SLP-65 thereby activating the signaling cascade for differentiation. In line with this, the B cell developmental block in mice with impaired PI3K signaling, p110dKO mice, is similar to that observed in the absence of BCAP/CD19 or in SLP-65-deficient mice^11,12,16^.

A recently identified patient lacking p85α showed a severe block in B cell development while other hematopoietic lineages were basically not affected^40^. Importantly, the phenotype of this p85α patient resembles that of λ5- or SLP-65-deficient patients. Both λ5 and SLP-65 represent well-known components of pre-BCR assembly or signaling^41,42^. In contrast, IL-7Rα-deficient patients lack T cells but have normal B-cell numbers^43,44^. If IL-7R signaling was essential for PI3K activation in developing B cells, PI3K-deficient patients were then expected to reveal a similar phenotype as IL-7R-deficient patients. Together, these findings support the view that PI3K activation is induced by pre-BCR signaling and that IL-7R is not essential for this process.

Notably, while B cell development is completely blocked in p110dKO mice, T cell development is not affected^24^. Moreover, our finding that p110dKO pre-B cells grow *in vitro* in IL-7 supplemented medium suggests that IL-7R dependent survival and proliferation signals do not require PI3K signaling. Together with our finding that p110dKO pre-B cells have reduced expression of Pax5 and SLP-65, the available data suggest that class IA PI3K activity is specifically important for the development of B cells.

The PI3K-mediated activation of Pax5 together with the well-established roles of PI3K signaling as key signaling axis for B cell survival and of Pax5 as essential factor for B cell development point to an unexpected mechanism, in which Pax5-mediated B cell differentiation is linked to survival. In this scenario, differentiation is a consequence of survival and proliferation. The molecular link between B cell survival and differentiation ensures that pre-BCR-mediated activation of class IA PI3K leads to B cell-specific gene expression and explains the specific importance of PI3K for B cell development. Our finding that PI3K is also required for Pax5 expression in mature B cells suggests that the BCR-generated survival signals, which are mediated by class IA PI3K^14^, maintain Pax5 expression at later developmental stages.

It should be noted, however, that certain amounts of Pax5 are expressed in developing cells before presence of μHC suggesting that other signaling pathways such as IL-7R are involved in the activation of Pax5 expression. IL-7R signals, which are known to activate survival and proliferation of pro-B cells^45^, may contribute to the induction of Pax5 expression by activation of STAT5 and Ebf1 that bind to the *Pax5* gene^46–49^. However, it is unlikely that IL-7R activates Pax5 expression by induction of PI3K signaling. In fact, no change in phosphorylation of AKT was detected upon ablation of *IL-7Rα* gene expression. Furthermore, the defective PI3K signaling in p110dKO mice led to a developmental block at the pre-B cell stage indicating that pro-B cells, which require IL-7 for survival, proceed normally through early developmental stages in the absence of PI3K. Thus, together with our results, the available data suggest that IL-7R might be involved in the induction of Pax5 expression by activation of STAT5 or Ebf1 but not by PI3K activation, which is specifically activated by the pre-BCR.

Notably, our study is in disagreement with previous findings suggesting that IL-7R signaling activates PI3K signaling and that attenuation of this IL-7R-induced PI3K-signaling is required for induction of Pax5 and thus pre-B cell differentiation^27^. However, the previous study mainly utilized Irf4/Irf8 double deficient pre-B cells and it is unclear how the absence of Irf4 influences the obtained results^27^. Since we identified Irf4 as critical regulator of Pax5 expression and Pax5 as important target for PI3K-mediated activation, it is conceivable that the lack of Irf4 may affect the regulation of PI3K signaling in the Irf4-deficient cells. Importantly, the ability of Irf4 to control Pax5 expression is supported by available reports showing that Irf4 represses *Pax5* gene expression^50^. In addition, available data show that the pre-BCR is only transiently expressed during early B cell development suggesting that its signaling activity is tightly regulated. Thus, experiments measuring induction of AKT phosphorylation after 4 days of reconstitution of pre-BCR expression in Rag2-deficent cells cannot detect any pAKT induction most likely because the transient pre-BCR signaling was terminated.

Our data suggest that the transient pre-BCR expression and function regulate the switch from proliferation to differentiation. During early phases of of pre-BCR signaling proliferation is induced and at the same time the expression of genes required for differentiation such as SLP-65 is activated. Induction of SLP-65 function downstream of the pre-BCR signaling down-regulates PI3K activity and allows differentiation. This scenario is in full agreement with our previous results elucidating the role of SLP-65^9^ and explains why deficiencies in Pax5, SLP-65, Irf4 or PI3K signaling are blocked at the pre-B cell stage of differentiation.

Moreover, we believe that characterization of the role of PI3K in regulating Pax5 expression and B cell commitment improves our understanding of general differentiation processes, as it elucidates how commitment, development and differentiation are regulated by a survival pathway such as PI3K, thereby maintaining the identity of the developing B cells. It is tempting to speculate that similar signaling networks exist in other hematopoietic lineages and might be exploited to regulate the differentiation, survival or proliferation of hematopoietic cells.

## Experimental Procedures

### Ethics statement

Wildtype (wt), SLP-65-deficient^11^, *FoxO1*^fl/fl^ ^51^, *p110α*^−/−^/*p110δ*^D910A^ ^24^, *p110α*^fl/fl^/*p110δ*^fl/fl^ ^52,53^, and *IL-7R*α^fl/fl^ ^28^ mice were used in this study. 8 to 10 weeks old Rag2/common γ chain double deficient mice^54^ were used for adoptive transfer of p110dKO cells. All experiments involving animals were reviewed and approved by the institutional animal care and use committee of Ulm University Medical Center and the Max-Planck-Institute of Immunobiology and Epigenetics. All methods were performed in compliance with the humane care and use of laboratory animals and the german animal welfare law. Buffy coats used for the preparation of human peripheral B cells were purchased from the Transfusion Center of Ulm University Medical Center (Institut für Klinische Transfusionsmedizin und Immungenetik Ulm GmbH, Ulm, Germany) and were obtained from anonymized healthy blood donors. All blood donors gave written informed consent to approve and authorize the use of their blood for medical, pharmaceutical, and research purposes.

### Cell culture and biochemistry

For generation of pro-/pre-B cell lines, we isolated bone marrow from the respective mice to generate IL-7-dependent pre-B cell lines. Freshly isolated murine cells were therefore cultured in Iscove’s medium containing 10% heat inactivated FCS (Vitromex), 2 mM L-glutamine, 100 U/ml penicillin, 100 U/ml streptomycin (Invitrogen), 5 × 10^−5^ M 2-mercaptoethanol and IL-7. IL-7 was produced in-house as a sterile-filtered supernatant of J558L cells. Culturing of primary bone marrow cells with medium containing IL-7 results in rapid enrichment of early B cells and finally in stably growing cell lines, which importantly can be propagated independent from feeder cell lines. This system to establish stable IL-7-dependent B cell cultures has been used by us and others extensively. Hallmark of this system is that all generated cell lines are dependent on exogenous IL-7 provided in cell culture medium. Consequently, withdrawal of IL-7 leads to rapid cell death of the cell lines (see Fig. 4a and data not shown). M-CSF was produced with help of the M-CSF producing L929S cell line (kindly provided by M. Freudenberg) or purchased from Immunotools. Mature B cells were isolated from mouse spleens by using negative selection with anti-CD43 MACS-beads (Miltenyi Biotec) and cultured in medium without IL-7. Human peripheral B cells (CD19^+^) were isolated by FACS sorting. The human pre-B cell line Nalm-6 and the mature B cell line SU-DHL-6 were cultured in medium without addition of IL-7. For inhibition of PI3K, SLP-65-deficient pre-B cells and bone marrow derived wt pre-B cells were treated with 30 μM LY294002 (Merck Biosciences), Nalm-6 and SU-DHL-6 cells with 50 μM LY294002 for the indicated time points. Freshly isolated primary mature murine B cells were treated with 15 μM LY294002 for the indicated time points. Stable growing cell lines of *FoxO1*^fl/fl^ (either IL-7 dependent or transformed by BCR-ABL) or *IL-7Rα*^fl/fl^ (IL-7 dependent) bm-derived pre-B cells were retrovirally transduced with tamoxifen-inducible Cre-recombinase (ER^T2^-Cre) or empty control vectors and subsequently selected by addition of puromycin. Cre-recombinase was activated by addition of 2 μM 4-hydroxytamoxifen (4-OHT). As a control cells were treated with EtOH (solvent of 4-OHT).

### PCR and RT-PCR

Total RNA was isolated from B cells using Trizol reagent (Invitrogen) or RNeasy Plus Mini KIT MiniPrep (Qiagen). The synthesis of cDNA was performed as previously described^39^. Gene expression was determined with gene specific primers using the SYBR-Green detection method (Applied Biosystems) and a 7500 Fast Real-Time PCR machine (Applied Biosystems). Results were calculated by the ∆C_T_-Method. Generally, sequences of all primers in this study are available upon request.

### Immunoblotting

For immunoblotting, 1.5 × 10^6^ cells per sample were harvested and lysed in 50 μl modified RIPA buffer (50 mM TrisHCl, pH 7.4, 1% NP-40, 0.25% sodium deoxycholate, 150 mM NaCl, 1 mM EDTA (pH 8), 1 mM sodium orthovanadate, 1 mM NaF and protease inhibitor cocktail (Sigma-Aldrich)). Lysates were separated on 10% SDS-polyacrylamide gels and transferred to PVDF membranes (Millipore). Membranes were blocked with 5% dry milk in PBT (PBS, 0.1% Tween-20) for 1 h at room temperature with constant agitation. Primary antibodies were diluted in PBT supplemented with 4% BSA fraction V (BIOMOL Research Laboratories). Secondary antibodies were diluted in blocking solution. Immunoreactive antibodies used were anti-Pax5 (Abcam), anti-FoxO1 (Cell Signaling Technologies), and anti-Actin (I-19, Santa Cruz Biotechnology).

### Flow Cytometry

Cells were surface-stained and analyzed by flow cytometry on LSRII flow cytometers (Becton Dickinson) using Cy5-, APC- or FITC-anti-IgM (μ-chain-specific, Southern Biotech), PE-anti-λ5 (LM34, a gift from A. G. Rolink), PE-Cy7-B220 (eBioscience), PE-B220 (BD Pharmingen) APC-CD19 (eBioscience), PE-CD43 (BD Pharmingen), PE-κ (Southern Biotech), PE-CD11b (eBioscience) and APC-CD127 (eBioscience). Intracellular stainings were performed using Fix and Perm cell permeabilization Kit (ADG) according to the manufacturer’s instructions. Antibodies used for intracellular FACS-staining were PE-anti-Pax5 (eBioscience), anti-SLP-65 (monoclonal IgG mouse, self-produced), anti-AKT (Cell Signaling), anti-FoxO1 (Cell Signaling), pAKT (Cell Signaling), APC-anti-rabbit IgG1 (AbD Serotec) and APC-anti-mouse IgG (AbD Serotec). PE-IgG2a κ (eBioscience), APC-IgG1 (eBioscience), APC-IgG2a κ (eBioscience) were used as isotype controls. PE-anti-Biotin (BioLegend), Alexa Fluor 647-anti-mouse-IgG Fab2 and Alexa Fluor 647-anti-rabbit-IgG Fab2 were used as secondary antibodies or as staining controls, respectively when used without the primary antibody.

### Plasmids and retroviral transduction

The plasmids for expression of the constitutively active mutant of AKT (myr-AKT), tamoxifen-inducible form of Cre (Cre-ER^T2^)^9^ and FoxO1-A3^55^ have been described previously. Open reading frames encoding for the constitutively active mutant of p110α (myr-p110α), Cre-recombinase and the human Irf4 (hIrf4) were subcloned into the pMIG-backbone containing an IRES-GFP cassette, thereby generating pMIG-myr-p110α, pMIG-Cre or pMIG-hIrf4-IRES-GFP, respectively. Target cells were retrovirally transduced as described previously^9^. In summary, the Phoenix retroviral producer cell line was transfected according to the manufacturer’s instructions using GeneJuice (Novagen). Retroviral supernatants were harvested after 36 and 60 h and for the subsequent transduction, pre-B cells were mixed with supernatants and centrifuged at 300 g at 37 °C for 3 h.

### Electroporation

WEHI cells were transfected with indicated constructs carrying upstream of Luciferase a Vκ21 promoter sequence as well as Renilla vector (rLuc) for normalization using Neon^TM^ transfection System (Lifetechnologies). Luciferase levels were detected by help of Dual Luciferase Assay system (Promega).

### Statistical analysis

P values < 0.05 were considered to be statistically significant (n. s. = not significant; *p < 0.05; **p < 0.01; ***p < 0.001; ****p < 0.0001).

The datasets generated and analyzed during the current study are available from the corresponding author on reasonable request.

## Electronic supplementary material

Supplementary Figures S1 - S6
