# Supplementary material for: PI3K induces B-cell development and regulates B cell identity
Source: Sci Rep. 2018 Jan 22;8:1327. doi: 10.1038/s41598-018-19460-5 (PMC5778048; doi:10.1038/s41598-018-19460-5)
Supplement: Supplementary file 1 — Supplementary Figures S1 - S6 [file 41598_2018_19460_MOESM1_ESM.pdf]

Supplementary information –

PI3K induces B-cell development and regulates B cell identity

Hend Abdelrasoul[1,2‡], Markus Werner[1‡], Corinna Setz[1], Klaus Okkenhaug[3], Hassan Jumaa[1*]

[1] Institute of Immunology; University Medical Center Ulm, 89081 Ulm, Germany

[2] Molecular Biology department, Genetic Engineering and Biotechnology Division, National Research Centre (NRC), 12622 Giza, Egypt

[3] Laboratory of Lymphocyte Signalling and Development, The Babraham Institute, Cambridge CB22 3AT, UK

‡ these authors contributed equally

Running title: PI3K activates Pax5 expression

Key words: PI3K, proliferation, B-cell development, differentiation, Pax5, FoxO1

*Correspondence to Hassan Jumaa: hassan.jumaa@uni-ulm.de

Figure S1. PI3K regulates Pax5 expression in B cells

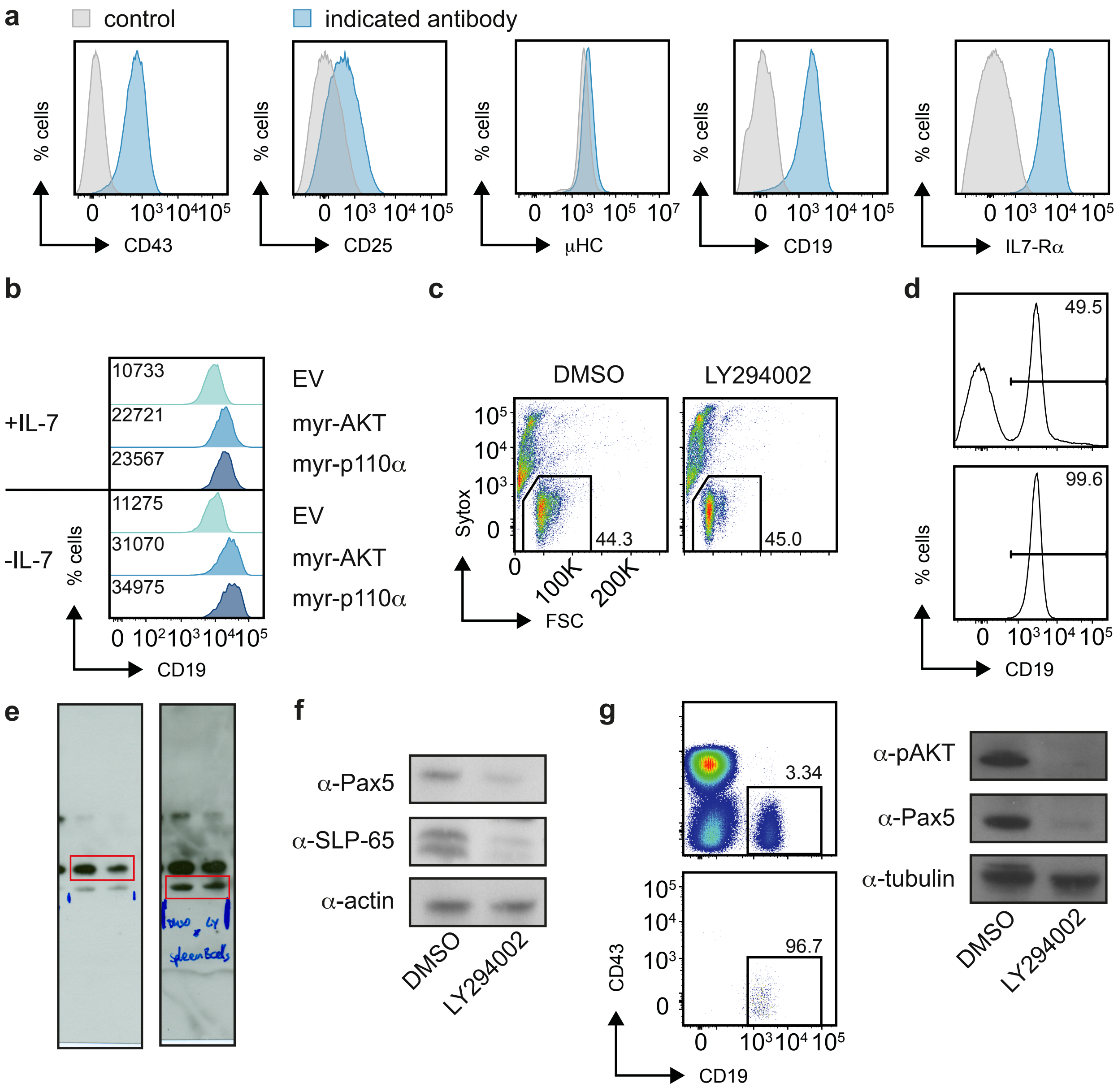

Supplementary Figure 1 (related to Fig. 1):

(**a**) Cells from a bone marrow (bm)-derived wildtype (wt) pre-B cell culture were characterized by flow cytometry for surface expression of CD43, CD25, Ig-µ heavy chain (µHC), CD19 and IL-7Rα. For CD25 and IL-7Rα cells were stained with respective isotype controls (gray), for CD43 and CD19 unstained cells were used as controls, whereas for the IgM staining, cells only stained with the secondary antibody served as control.

(**b**) Cells from a bm-derived wt pre-B cell culture were transduced with constitutively active forms of AKT (myr-AKT), p110α (myr-p110α), or as control with empty vector (EV) and cultured for three days in presence or absence of IL-7 before analysis of CD19 surface expression by FACS. Numbers in histograms indicate the MFI of the GFP$^+$ populations.

(**c**) Assessment of viability in cells from a bm-derived wt pre-B cell line after 16 h of treatment with the PI3K-inhibitor LY294002 (15 µM). Numbers in the dot plots indicate the percentages of viable cells.

(**d**) Murine mature primary B cells were purified by magnetic activated cell sorting and the percentages of CD19$^+$ cells were assessed by flow cytometry prior to (top) and after isolation (bottom).

(**e**) Murine mature primary B cells (CD43$^-$) were isolated and treated with LY294002 or DMSO for 12 h, lysed and subjected to immunoblot for analysis of Pax5 expression. Original full-lenght blots shown in Fig. 1d: The upper two bands of the left blot were selected to show Pax5 expression, whereas the lower bands in the right blot (longer exposure time) were cropped to show actin (loading control). Cropped areas are indicated by red squares.

(**f**) Inhibition of PI3K decreases Pax5 and SLP-65 expression in human B cells. Human pre-B cells were treated with LY294002 or the solvent DMSO for 12 h. Levels of Pax5 and SLP-65 were assessed by immunoblotting. Actin served as loading control.

(**g**) Human mature B cells were FACS-purified according to the gating strategy (left panel, top) and purity was confirmed by reanalysis (left panel, bottom). Cells were treated with LY294002 and levels of pAKT and Pax5 were assessed by immunoblotting (right panel). Tubulin served as loading control.

Figure S2. Controls for deletion of *IL-7Rα*$^{fl/fl}$ and *FoxO1*$^{fl/fl}$

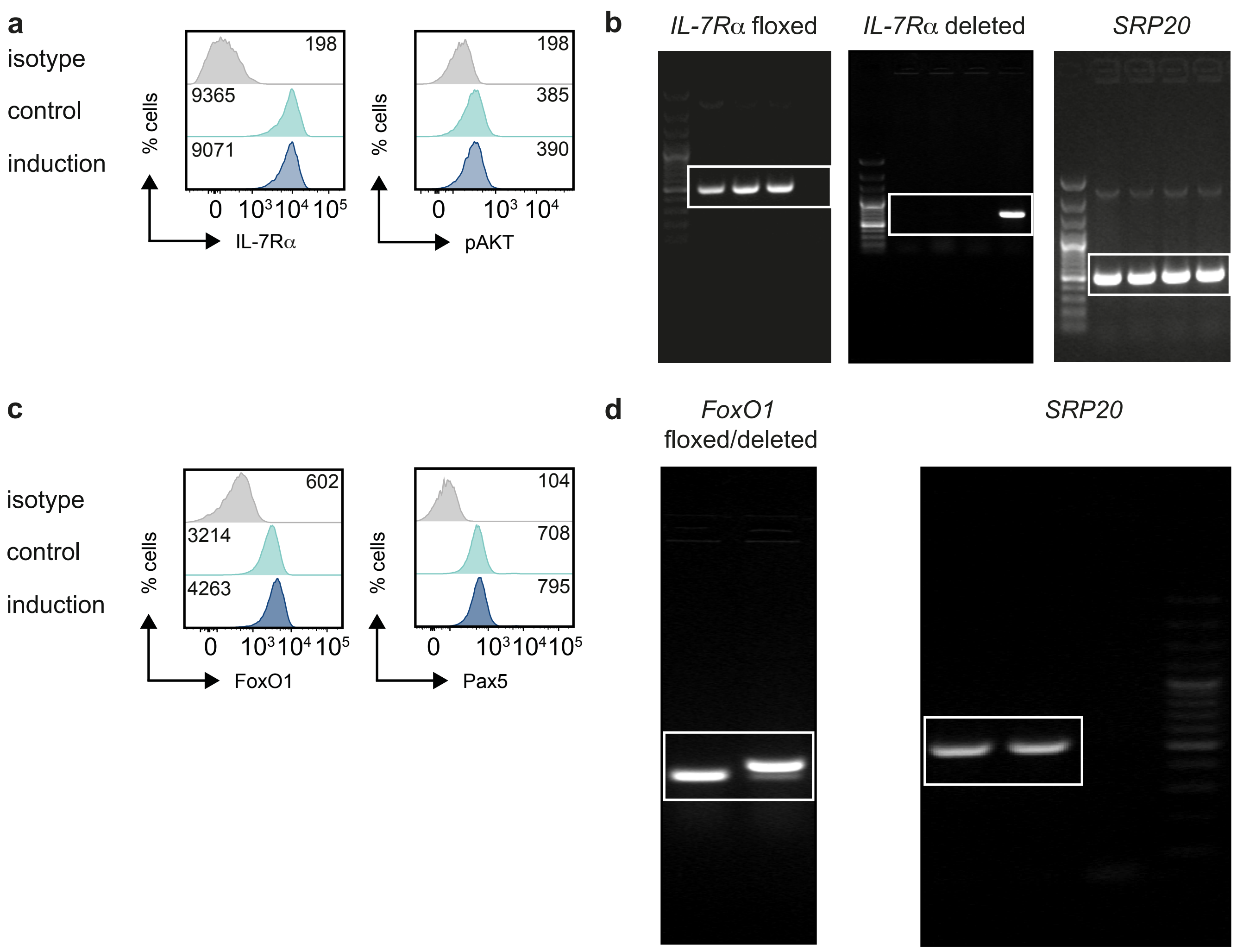

Supplementary Figure 2 (a-b related to Fig. 2 and c-d related to Fig. 5):
(**a**) *IL-7Rα*$^{fl/fl}$ cells were transduced with ER^{T2} and treated with 4-hydroxytamoxifen (4-OHT) or EtOH for 48 h, respectively. Surface expression of IL-7Rα and intracellular levels of pAKT were determined by flow cytometry.
(**b**) Original full-length gel pictures used to show the Cre-mediated *IL-7Rα* deletion by PCR in Fig. 2b. Cropped regions are indicated by white squares.
(**c**) *FoxO1*$^{fl/fl}$ cells were transduced with ER^{T2} and treated with 4-OHT or EtOH for 48 h, respectively. Intracellular levels of FoxO1 and Pax5 were determined by FACS.
(**d**) Original full-length gel pictures used to show the Cre-mediated *FoxO1* deletion in Fig. 5d. Cropped regions are indicated by white squares.

Figure S3. PI3K regulates Pax5 expression in B cells

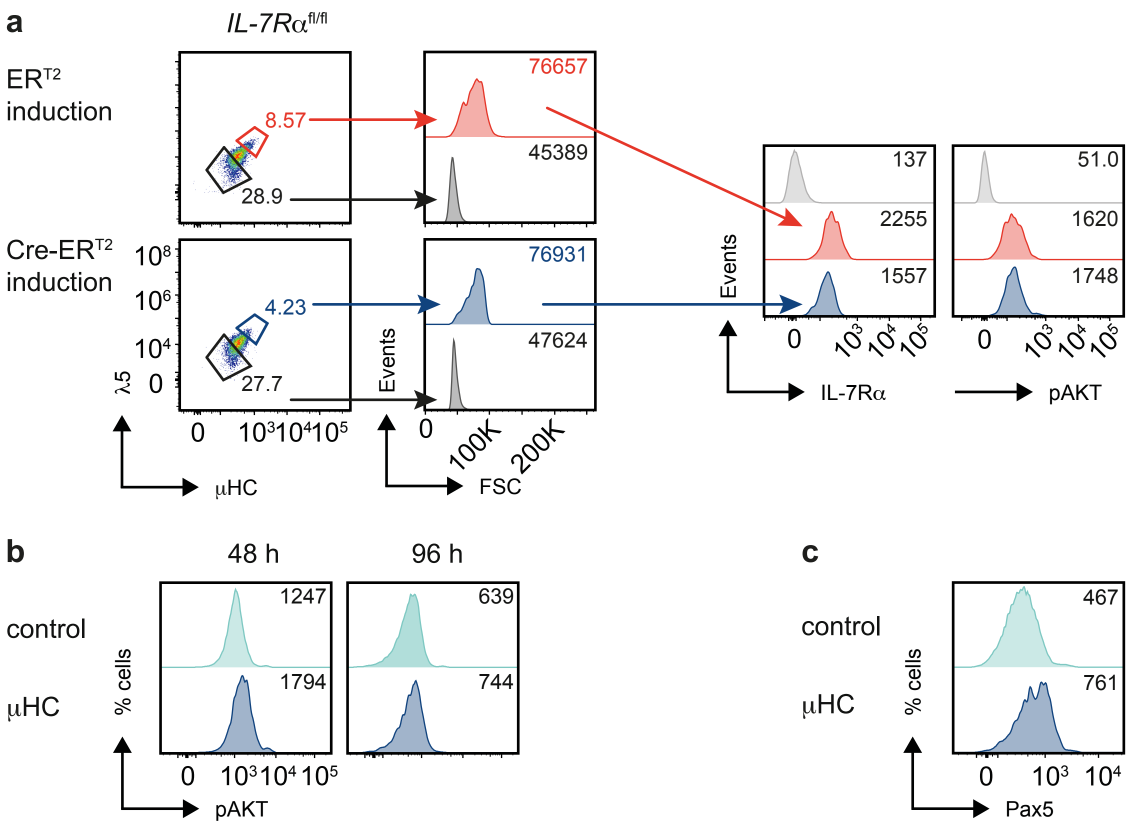

Supplementary Figure 3 (related to Fig. 2):
(**a**) Primary bm-derived *IL-7R*α$^{fl/fl}$ B cells were transduced either with ER^{T2} or Cre-ER^{T2} and induced with 4-OHT for 24 h, respectively. Cells were stained at the surface for IL-7Rα, then permeabilized and stained intracellularly for λ5, μHC and pAKT. Small and large pre-B cells were distinguished by expression of the pre-BCR (μHC$^+$/λ5$^+$) and forward scatter (FSC). IL-7Rα expression and AKT phosphorylation were compared in the fraction of large pre-B cells from induced IL-7Rα$^{f/f}$-ER^{T2} and -Cre-ER^{T2} cells.
(**b**) Cells from a Rag2-deficient B cell culture were retrovirally transduced with μHC (B1-8 HC). Levels of pAKT were measured by FACS at 48 (day 2) and 96 h (day 4) upon transduction and compared to the expression in untransduced bystander cells (control). Numbers in the histogram plots indicate the MFI.
(**c**) Pax5 expression was measured by FACS in the cells described in Fig. S3b at 48 h (day 2) following transduction and compared to the levels measured in untransduced bystander cells (control). Numbers in the histogram plots indicate the MFI.

Figure S4. Characterization of p110dKO cells

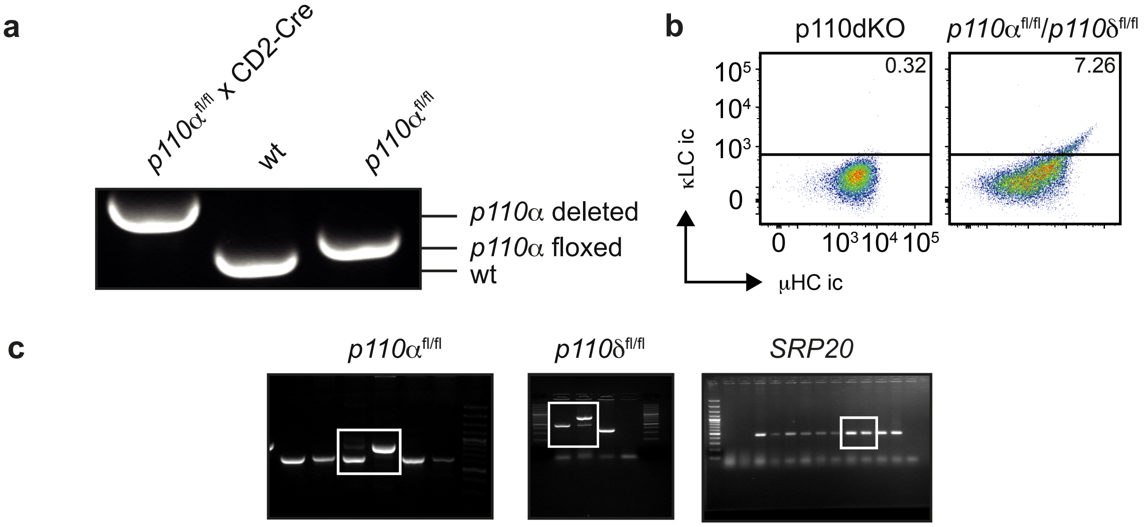

Supplementary Figure 4 (related to Fig.3):

(**a**) Verification of the *p110*α deletion by CD2-cre in p110dKO cells. *p110*δ inactivation results from the D910A mutation whereas the floxed *p110*α allele can be deleted by CD2-cre.

(**b**) p110dKO and *p110*α$^{fl/fl}$/*p110*δ$^{fl/fl}$ cells were stained for intracellular (ic) expression of Ig-κ light (κLC) and μ-heavy chain (μHC). While *p110*α$^{fl/fl}$/*p110*δ$^{fl/fl}$ cells behave similar to wt, p110dKO cells are blocked in differentiation.

(**c**) Original full-length gel pictures used to show the Cre-mediated deletion of *p110*α and *p110*δ in Fig. 3g. Cropped regions are highlighted by white squares.

Figure S5. Injection of p110dKO cells into Rag-2/γc double deficient mice

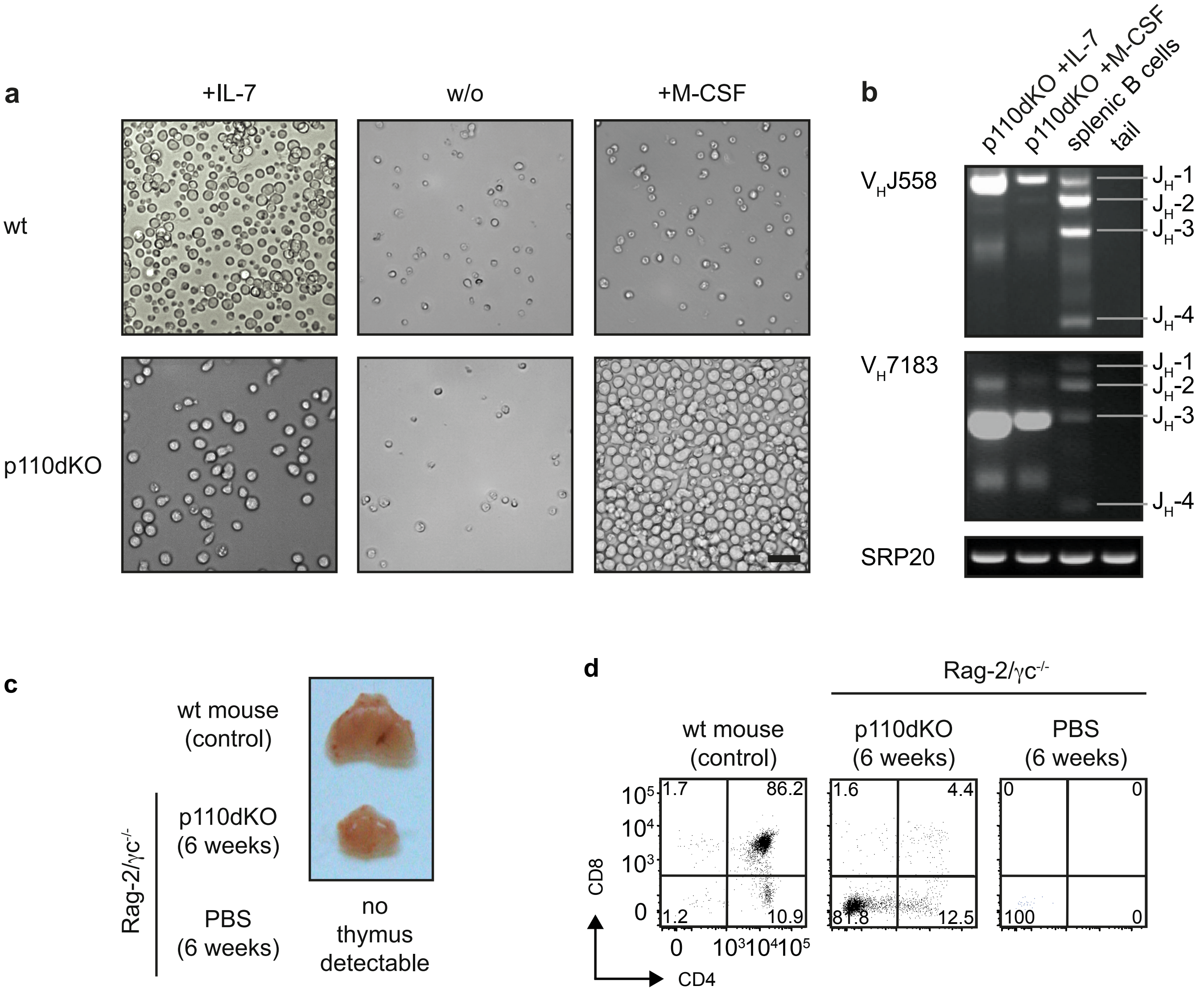

Supplementary Figure 5 (related to Fig. 4):

(**a**) Representative pictures from wt and p110dKO cells cultured in medium supplemented with IL-7 (+IL-7), M-CSF (+M-CSF) or in absence of cytokines for 7 days. Scale bar represents 25 μm.

(**b**) Analysis of VDJ recombination at the IgH locus in gDNA from p110dKO cells cultured either in presence of IL-7 or M-CSF in comparison to gDNA from wt splenic B cells or tail. For detection of recombinations involving proximal V_H gene segments the forward primer is located in V_H7183 whereas for distal V_H gene segments it is located within V_HJ558. The corresponding reverse primer is located downstream of J_H4.

(**c**) Macroscopic appearance of thymi from Rag-2/γc double deficient mice injected with p110dKO cells (n = 5) and cells from a wildtype (wt) mouse as control (n = 5). No thymus was detectable in Rag-2/γc double deficient mice injected with PBS (n = 3). Cells were injected into 8 - 10 weeks old Rag-2/γc double deficient mice which were analyzed 6 weeks after injection.

(**d**) FACS-analysis of cells from thymi shown in Fig. S5c stained for CD4 and CD8 (gated on $CD19^-$ and (ic) μHC^- cells). Numbers in quadrants indicate percentages of cells in the respective gates.

Figure S6. Irf8 does not affect Pax5 expression

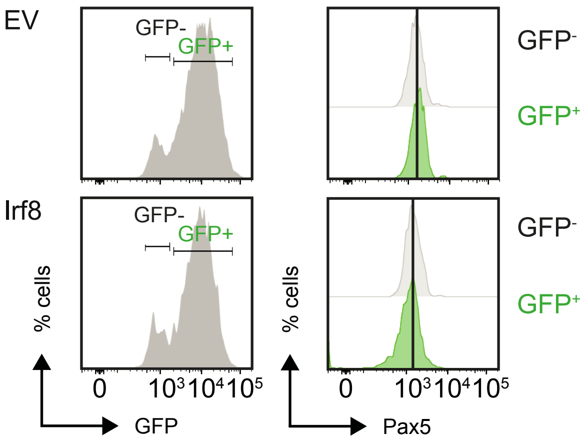

Supplementary Figure 6 (related to Fig. 6):
Irf8 does not affect Pax5 expression. Cells from a bm-derived wt pre-B cell line were transduced with a vector encoding Irf8 containing in addition IRES-GFP (Irf8) or an empty control vector containg only IRES-GFP (EV). At day 2 after transduction levels of Pax5 were analyzed within the respective GFP^+ and GFP^- populations of both experimental arms by FACS.
